# The Antimicrobial Resistance of Enterotoxigenic *Escherichia coli* from Diarrheal Patients and the Environment in Two Geographically Distinct Rural Areas in Bangladesh over the Years

**DOI:** 10.3390/microorganisms12020301

**Published:** 2024-01-31

**Authors:** Fatema-Tuz Johura, Marzia Sultana, Abdus Sadique, Shirajum Monira, David A. Sack, Richard Bradley Sack, Munirul Alam, Subhra Chakraborty

**Affiliations:** 1 International Centre for Diarrhoeal Disease Research, Bangladesh, Dhaka 1212, Bangladesh; mjohura1@jh.edu (F.-T.J.); msultana@icddrb.org (M.S.); sadique004@gmail.com (A.S.); smonira@icddrb.org (S.M.); munirul@icddrb.org (M.A.); 2 Department of International Health, Johns Hopkins Bloomberg School of Public Health, Johns Hopkins University, Baltimore, MD 21205, USA; dsack1@jhu.edu (D.A.S.); rsack@jhsph.edu (R.B.S.)

**Keywords:** antibiotic resistance, ETEC, multi-drug resistant

## Abstract

Antimicrobial resistance (AMR) is an unprecedented global health challenge, involving the transfer of bacteria and genes between humans and the environment. We simultaneously and longitudinally determined the AMR of enterotoxigenic *Escherichia coli* (ETEC) strains isolated from diarrheal patients and an aquatic environment over two years from two geographically distinct locations, Coastal Mathbaria and Northern Chhatak in Bangladesh. A total of 60% and 72% of ETEC strains from the patients in Mathbaria and Chhatak, respectively, were multi-drug resistant (MDR) with a high proportion of ETEC resistant to nalidixic acid (80.7%), macrolides (49.1–89.7%), ampicillin (57.9–69%), and trimethoprim/sulfamethoxazole (55.2%). From the surface water, 68.8% and 30% of ETEC were MDR in Mathbaria and Chhatak, respectively, with a high proportion of ETEC strains resistant to macrolides (87.5–100%), ampicillin (50–75%), ceftriaxone (62.5%), and nalidixic acid (40%). Notably, 80–100% of the ETEC strains were susceptible to tetracycline and quinolones (ciprofloxacin and norfloxacin), both in clinical and aquatic ETEC. The AMR varied by the ETEC toxin types. The patterns of excessive or limited consumption of drugs to treat diarrhea over time in Bangladesh were reflected in the ETEC AMR from the patients and the environment. The high prevalence of MDR-ETEC strains in humans and the environment is of concern, which calls for vaccines and other preventative measures against ETEC.

## 1. Introduction

Enterotoxigenic *E*. *coli* (ETEC) is a leading cause of diarrheal morbidity and mortality in low- and middle-income countries (LMICs) and is diagnosed less frequently than its occurrence [1,2]. ETEC is often the first bacterial pathogen that causes diarrhea in neonates and children and is responsible for two to five diarrheal episodes during the first 3 years of life [3]. It is estimated that ETEC causes about 220 million diarrhea episodes globally, with about 75 million episodes in children under 5 years of age, resulting in between 18,700 and 42,000 deaths [4]. In addition, ETEC is a significant pathogen causing diarrheal episodes in travelers and in militaries from high-income countries who visit LMICs [5].

According to the WHO guidelines, antibiotics should be used for the cases of cholera and cases with high fever and dysentery (visible blood in stool), the majority of which is caused by *Shigella* spp. [6]. Therefore, antibiotics are not recommended for ETEC diarrhea for people in LMICs. ETEC causes watery diarrhea, which occurs between 8 and 72 h after initial infection, usually following the ingestion of contaminated food or water [1]. The severity of disease varies from a mild illness to severe disease. Most cases of ETEC diarrhea are self-limiting and do not require antibiotic treatment, but treatment is often given empirically depending on the severity of the disease and the risk of complications. Children with nutritional deficiencies or medical comorbidities may have more severe presentations and require the use of antimicrobial agents [7]. Patients with persistent diarrhea due to ETEC also may benefit from antibiotic therapy [8].

When ETEC was first identified, the bacterium was highly sensitive to all relevant antimicrobials, including tetracyclines and trimethoprim/sulfamethoxazole [9]. Antimicrobials that have been used for prophylaxis for traveler’s diarrhea include doxycycline, ciprofloxacin, and rifaximin [10,11,12,13]. Others have been used for treatment including quinolones, azithromycin, cotrimoxazole, and rifaximin [14]. Fluoroquinolone-resistant *E. coli* has been increasingly reported during the last decade in both the hospital environment and the community, which has limited the utility of these broad-spectrum agents [15]. The antimicrobial treatment of traveler’s diarrhea has altered over the years due to increasing antimicrobial resistance [14]. In the last decade, resistance against commonly used drugs for the treatment of enteric infections in travelers, including ampicillin, tetracycline, and cotrimoxazole, has increased among diarrheagenic *E. coli* [16]. There are no data available on the antibiotic resistance of ETEC isolated from environmental sources. Monitoring AMR in ETEC simultaneously from the patients and environment over the years is required to understand the current prevalence and changing patterns of AMR. Understanding transmission between humans and the environment will facilitate planning strategies to control this significant public health problem.

We conducted a study from 2014 to 2016 in Bangladesh to determine the prevalence and characterizations of ETEC among patients with diarrhea who sought care at health facilities [2] and simultaneously from the aquatic bodies that are used by the patients for their daily activities like drinking water, bathing, washing clothes and utensils, etc. In this current study, we used the ETEC strains that were isolated in a parent surveillance study and determined the AMR of ETEC causing diarrhea in patients and from ETEC from the aquatic environment, as well as the longitudinal changes over the years in two geographically distinct rural areas, Mathbaria in the southern coast and Chhatak in the north of Bangladesh. We also determined if the AMR patterns relate to the toxin types of ETEC.

## 2. Materials and Methods

Study sites: The two study sites selected for this study were geographically different. Mathbaria is in the southern coastal area located adjacent to the Bay of Bengal, approximately 400 km southwest of Dhaka (the capital city of Bangladesh), and Chhatak is a northern landlocked hilly area in the northeastern part of Bangladesh, which is in the Sunamganj district, 264 km northeast of Dhaka. From both study sites, environmental samples were collected from rivers, ponds, and canals. Rectal swabs or stool samples were collected from diarrheal patients from the Thana Health Complexes (THCs) in each site, which are government rural healthcare facilities.

### 2.1. Sample Collection and Processing in the Surveillance Study

In this study, stool and rectal swab samples from Mathbaria and Chhatak were collected weekly during a diarrhea peak season and monthly during the diarrhea off-peak season from patients attending the THCs [2]. Environmental samples were collected simultaneously with the stool samples from the water bodies that were commonly used by the residents for daily activities.

Stool Samples: Rectal swabs in 2014 (April–August) and stool samples between 2015 (April–November) and 2016 (May) were collected from patients with diarrhea who sought care at the Mathbaria health complex [2]. Rectal swabs were collected from the patients with diarrhea at the Chhatak health complex in 2014 [2]. The rectal swabs were placed in a Cary–Blair medium, and stool sample cups were placed in a cool pack [17,18] immediately after collection and transported to the central laboratory of the International Center for Diarrheal Disease Research, Bangladesh (ICDDR,B), in Dhaka. In the laboratory, the rectal swab and stool samples were inoculated directly onto a MacConkey agar plate and incubated at 37 °C for 16–18 h. From each sample, 5 lactose-fermenting presumptive E. coli colonies were selected from MacConkey agar plates and tested for ETEC toxin genes, LT, STh, and STp, using PCR as described before [2].

Environmental samples: Surface water, plankton, and sediment samples were collected from rivers, ponds, and canals between 2015 (March–December) and 2016 (April–May) from Mathbaria and in 2014 (April–November) from Chhatak. In Mathbaria, we also collected community-common source drinking water from the pond sand filters (PSF), where a hand pump was attached to a sand filter, which collected water from a pond with the aim of purifying the pond water. All the samples were collected aseptically in sterile glass bottles, transported to the laboratory, and preserved in an ambient temperature. After receiving all the samples at the ICDDR,B laboratory, the water samples were filtered through a 0.22 µm bacteriological membrane filter (MilliporeCorp., Bedford, MA, USA) and were processed as previously reported [19]. Plankton samples were concentrated by filtering through a 20 µm mesh nylon filter and homogenizing in a Teflon-tipped tissue grinder (Wheaton Scientific, Millville, NJ, USA) [19]. Ten grams of sediment was vortexed in a 90 mL sterile physiological saline for 2 min [19].

After processing, all the samples were enriched in a MacConkey broth at 37 °C for 18–24 h before plating. About 100 µL of enriched broth was serially diluted and spread using a sterile spreader onto a MacConkey agar plate and incubated at 37 °C for 16–18 h. From each sample, 50 isolated lactose-fermenting *E. coli*-like colonies were selected for testing ETEC toxin genes via PCR [2].

### 2.2. Antibiotic Susceptibility Testing

In this current study, we analyzed the antimicrobial susceptibility pattern of ETEC isolates. ETEC isolates were subjected to an antimicrobial susceptibility test against eleven antibiotics that belong to seven different antibiotic groups using the Kirby–Bauer disc diffusion method [20] following the Clinical and Laboratory Standards Institute (CLSI) guidelines [21]. The following antibiotics were chosen based on their prevalent local usage: quinolone [nalidixic acid (NA: 30 µg), ciprofloxacin (CIP; 5 µg), norfloxacin (NOR; 10 µg)], tetracyclines [tetracycline (TE; 30 µg), doxycycline (D; 30 µg)], macrolide [azithromycin (AZM; 15 µg), erythromycin (E; 15 µg)], trimethoprim/sulfamethoxazole (SXT; 25 µg), ceftriaxone (CRO; 30 µg), ampicillin (AMP; 10 µg), and streptomycin (S; 15 µg). All the antibiotic discs, except doxycycline (D; 30 µg), were procured from Oxoid Limited (Hampshire, UK). Doxycycline was purchased from BD, USA. After 24 h of incubation of the ETEC culture on Mueller–Hinton agar plates, the diameter of the zone of inhibition was measured in millimeters (mm), and the sensitivity pattern of each isolate was recorded according to the inhibition zone size scale provided by the CLSI standards.

We also tested ETEC-negative *E. coli* (non-ETEC) isolates from eighteen diarrhea patients and five environmental samples from Mathbaria and Chhatak to compare their antibiotic resistance with the ETEC isolates.

### 2.3. Data Analysis

Data analysis was done using Microsoft 365 Excel (Version 2312 Build 16.0.17126.20132) 64-bit. Due to the small sample size, this study lacks statistical power.

### 2.4. Ethics Statement

This study was approved by the institutional review boards of the ICDDR,B and Johns Hopkins University. Written informed consent was obtained from the adults and the caregivers of the children who participated in this study.

## 3. Results

Using PCR, we detected ETEC-specific virulence genes LT, STh, and STp from the samples collected throughout the study period from Mathbaria and Chhatak [2]. In this current study, we tested the AMR patterns on 124 ETEC isolates from Mathbaria and Chhatak from patient samples and from the environmental samples as is shown in Table 1.

### 3.1. AMR in the ETEC Strains Isolated from Diarrheal Patients

**Figure 1:**
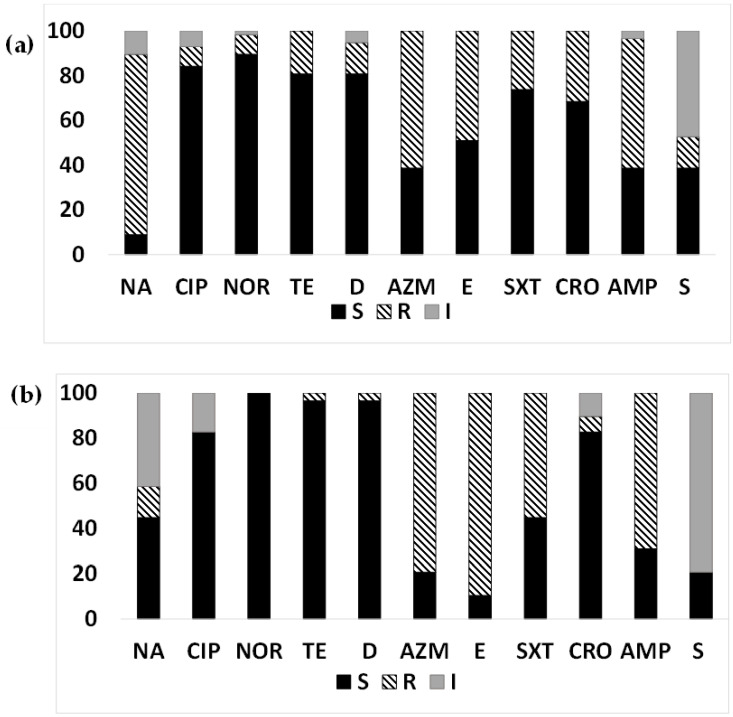
Antimicrobial susceptibility pattern of ETEC from diarrheal patients: Antimicrobial susceptibility pattern (the proportion of sensitive or resistant strains) of ETEC strains isolated from diarrheal patients in Mathbaria and Chhatak. (**a**) AMR pattern among ETEC strains in Mathbaria isolated in 2014–2016, (**b**) AMR pattern among ETEC strains in Chhatak isolated in 2014. Black-filled bar: sensitive; Pattern-filled bar: Resistant; and Grey-filled bar: intermediate susceptibility. The x axis represents the antibiotic, and the y axis represents the percentage of sensitive and resistant strains.

Among the ETEC strains isolated from diarrheal patients (Figure 1a,b), most ETEC strains were susceptible to tetracyclines (80.7% in Mathbaria and 96.6% in Chhatak) and to quinolones Mathbaria (84.21–89.47%) and Chhatak (82.76–100%), except more isolates were resistant to nalidixic acid (80.7% in Mathbaria compared to 13.8% resistant and 41.4% intermediate resistance in Chhatak). Resistance to macrolides, both azithromycin and erythromycin, were very high in both sites, 79.31–89.66% in Chhatak and 49.1–61.4% in Mathbaria. A majority of the ETEC strains from Chhatak showed resistance to ampicillin (68.9%) and trimethoprim/sulfamethoxazole (55.17%) compared to Mathbaria (ampicillin, 57.9%; and trimethoprim/sulfamethoxazole, 26.3%). More ETEC strains from Mathbaria were resistant to ceftriaxone (31.58%) compared to Chhatak (6.9%). Intermediate susceptibility to streptomycin was found among the ETEC strains from Chhatak (79.31%) compared to Mathbaria (47.37%).

### 3.2. MDR-ETEC from Diarrheal Patients

Among the ETEC strains isolated from diarrheal patients in Mathbaria, 59.7% were multi-drug resistant (MDR). MDR-ETEC strains showed nine different resistance profiles (Table 2) with the highest resistance towards six antibiotic groups. The most prevalent profile among the diarrhea patients with MDR-ETEC was quinolone, macrolide, and ampicillin (29.41%). Among the ETEC strains isolated from diarrheal patients in Chhatak, 72.41% were multi-drug resistant. The MDR-ETEC strains from Chhatak showed four different resistance profiles (Table 2), with the highest resistance towards three antibiotic groups. The most prevalent profile among the Chhatak MDR-ETEC strains was macrolide, trimethoprim/sulfamethoxazole, and ampicillin.

### 3.3. AMR Pattern of ETEC Strains Based on Toxin Gene Profiles Isolated from Diarrheal Patients

**Figure 2:**
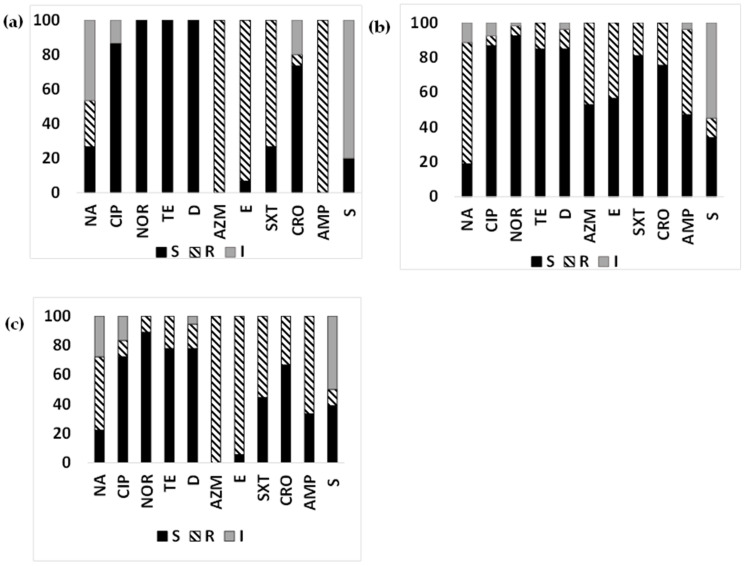
Antimicrobial susceptibility pattern of diarrheal ETEC based on toxin gene types: Antimicrobial susceptibility pattern (the proportion of sensitive or resistant strains) of ETEC based on toxin gene types (LT and ST) isolated from diarrheal patients of Chhatak and Mathbaria. (**a**) AMR pattern of LT-ETEC strains; (**b**) AMR pattern of ST-ETEC; and (**c**) AMR pattern of LT+ST-ETEC. Black-filled bar: sensitive; Pattern-filled bar: Resistant; and Grey-filled bar: intermediate susceptibility. The x axis represents the antibiotic, and the y axis represents the percentage of sensitive and resistant strains.

We further analyzed if the susceptibility patterns of the ETEC strains isolated from the patients varied by the types (toxin genes) of ETEC (Figure 2a–c). While a higher proportion of ST-ETEC (69.8%) and LT+ST-ETEC (50%) were resistant to nalidixic acid compared to LT-ETEC (26.67%), resistance profiles to ciprofloxacin and norfloxacin were similar among the ETEC toxin types. Notably, almost all the LT-ETEC and LT+ST-ETEC (93.3–100%), and about 50% (43.4–47.2%) of the ST-ETEC, were resistant to macrolides. While 11.3–22.2% of the LT+ST-ETEC and ST-ETEC strains were resistant to tetracyclines, all the LT-ETEC strains were susceptible. Trimethoprim/sulfamethoxazole resistance was highest among the LT-ETEC profiles (73.3%), followed by LT+ST-ETEC (55.6%) and ST-ETEC (18.87%). The highest number of ceftriaxone resistance was found among LT+ST-ETEC (33.3%), followed by ST-ETEC (24.6%) and LT-ETEC- (6.67%). While LT-ETEC- strains were 100% resistant to ampicillin, 66.7% of the LT+ST-ETEC and about half of the ST-ETEC strains were resistant. LT-ETEC (80%) showed highest intermediate susceptibility to streptomycin, followed by ST-ETEC (54.72%) and LT+ST-ETEC (50%).

### 3.4. AMR in the ETEC Strains from the Aquatic Environment

**Figure 3:**
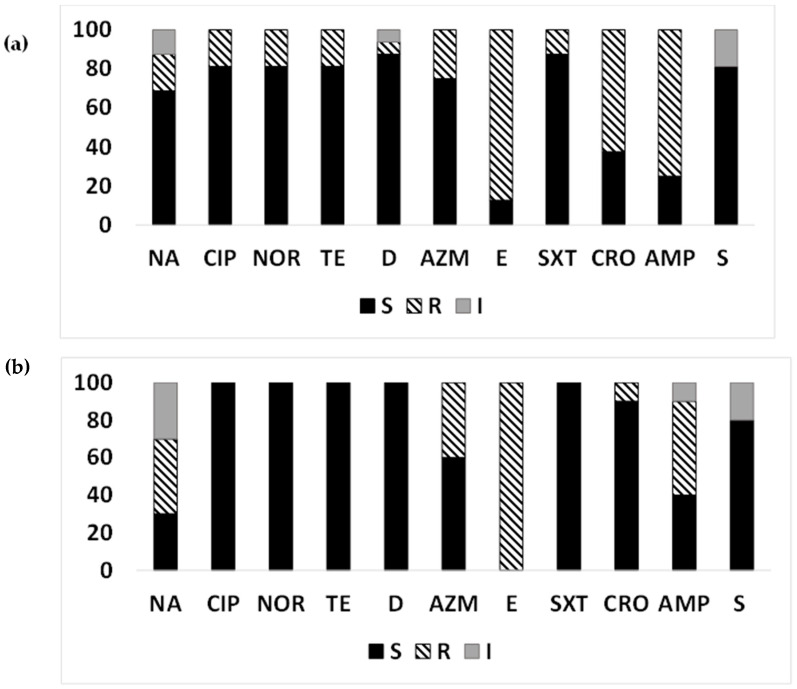
Antimicrobial susceptibility pattern of ETEC from surface water: Antimicrobial susceptibility pattern (the proportion of sensitive or resistant strains) of ETEC strains isolated from surface water in Mathbaria and Chhatak. (**a**) AMR pattern among ETEC strains in Mathbaria in 2015–2016; (**b**) AMR pattern among ETEC strains in Chhatak in 2014. Black-filled bar: sensitive; Pattern-filled bar: Resistant; and Grey-filled bar: intermediate susceptibility. The x axis represents the antibiotic, and the y axis represents the percentage of sensitive and resistant strains.

Among the quinolones, the proportion of the ETEC strains resistant to nalidixic acid was higher—40% in Chhatak and 18.75% in Mathbaria—but the proportion resistant to ciprofloxacin and norfloxacin was only 18.75% in Mathbaria, and all the strains from Chhatak were susceptible (Figure 3a,b). Among the macrolides, a higher proportion of ETEC strains was resistant to erythromycin (100% in Chhatak and 87.5% in Mathbaria), compared to azithromycin (40% in Chhatak and 25% in Mathbaria). While none of the environmental ETEC strains were resistant to tetracycline and trimethoprim/sulfamethoxazole from Chhatak, in Mathbaria, 6.25–18.75% were resistant to tetracyclines, and 12.5% strains were resistant to trimethoprim/sulfamethoxazole. Ceftriaxone- and ampicillin-resistant ETEC were found in higher proportions in Mathbaria (62.5% and 75%, respectively) than Chhatak (10% and 50%, respectively). Intermediate susceptibility was observed for the streptomycin group of antibiotics in both sites, Chhatak (20%) and Mathbaria (18.75%).

Among the ETEC strains isolated from sediment, resistance was observed for macrolides (33.33–100%), followed by ceftriaxone, ampicillin (66.67%), and nalidixic acid (33.33%). Among the ETEC isolates from plankton, the highest resistance was observed for macrolides (50–100%), followed by ampicillin (50%).

### 3.5. MDR-ETEC from Surface Water

Among the ETEC strains isolated from Mathbaria surface water, 68.75% were multi-drug resistant. MDR-ETEC strains showed five resistance profiles (Table 3) with the highest resistance towards four antibiotic groups. The most prevalent profile among Mathbaria surface water MDR-ETEC was macrolide, ceftriaxone, and ampicillin (63.6%). Among the ETEC strains isolated from Chhatak surface water, 30% were multi-drug resistant. MDR-ETEC showed two resistance profiles with highest resistance towards three antibiotic groups. The most prevalent profile among the MDR-ETEC isolated from Chhatak surface water was quinolone, macrolide, and ampicillin (66.67%) (Table 3).

### 3.6. AMR Pattern of ETEC Strains Based on Toxin Gene Profiles Isolated from Surface Water

**Figure 4:**
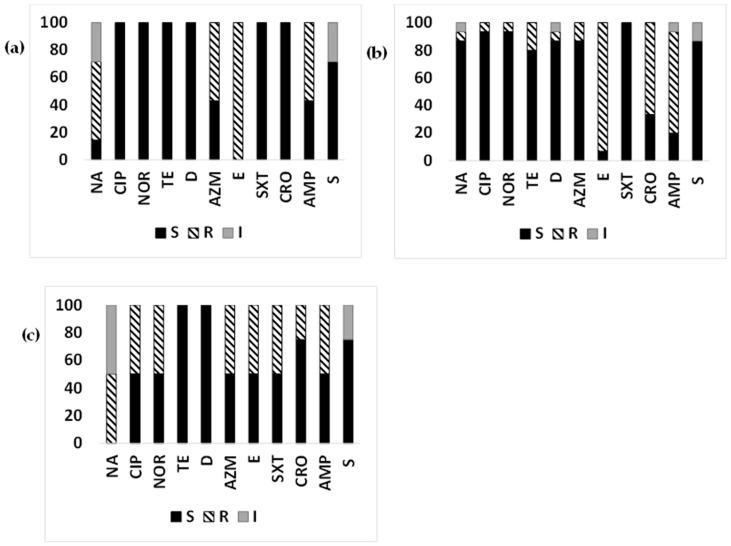
Antimicrobial susceptibility pattern of surface-water ETEC based on toxin gene types: Antimicrobial susceptibility pattern (the proportion of sensitive or resistant strains) of ETEC strains based on toxin gene types (LT and ST) isolated from surface water. (**a**) The AMR pattern of LT-ETEC isolated from the surface water of Chhatak; (**b**) the AMR pattern of ST-ETEC strains isolated from the surface water of Chhatak and Mathbaria; (**c**) the AMR pattern of ST+LT-ETEC isolated from the surface water of Mathbaria. Black-filled bar: sensitive; Pattern-filled bar: Resistant; and Grey-filled bar: intermediate susceptibility. The x axis represents the antibiotic, and the y axis represents the percentage of sensitive and resistant strains.

Among the ETEC strains isolated from surface water in Mathbaria and Chhatak, LT-ETEC (57.14%) and LT+ST-ETEC had higher resistance to nalidixic acid compared to ST-ETEC (6.67%) (Figure 4a–c). LT+ST-ETEC showed the highest resistance to other quinolones (50%) compared to ST-ETEC (6.67%), while all LT-ETEC profiles were susceptible. Among the macrolides, erythromycin resistance was higher in LT-ETEC (100%), ST-ETEC (93.33%), and LT+ST-ETEC (50%), compared to azithromycin (13.33–57.14%). While 6.67–20% of ST-ETEC strains were resistant to tetracyclines, all the LT-ETEC and LT+ST-ETEC strains were susceptible. Trimethoprim/sulfamethoxazole-resistant strains were observed among LT+ST-ETEC (50%), while all the LT-ETEC and ST-ETEC strains were susceptible. The highest number of ceftriaxone-resistant strains were found among ST-ETEC (66.67%), followed by LT+ST-ETEC (50%), but all the LT-ETEC isolates were susceptible. Most ST-ETEC (73.33%) were resistant to ampicillin, followed by LT-ETEC (57.14%) and LT+ST-ETEC (50%). LT-ETEC (28.57%) showed intermediate susceptibility to streptomycin, followed by LT+ST-ETEC (25%) and ST-ETEC (13.33%) (Figure 4a–c).

### 3.7. Year-Wise AMR Pattern of ETEC Strains

**Figure 5:**
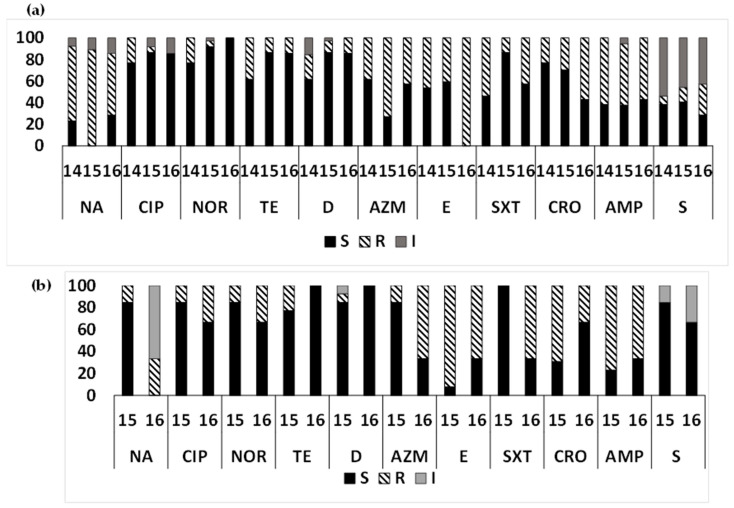
Year-wise antimicrobial susceptibility pattern of ETEC strains: The proportion of sensitive or resistant (**a**) ETEC strains isolated from diarrheal patients in Mathbaria in 2014–2016; (**b**) ETEC strains isolated from surface water in Mathbaria in 2015–2016.

Year-wise susceptibility analysis (Figure 5a) of ETEC strains from clinical cases from Mathbaria from 2014 to June 2016 suggested that nalidixic acid resistance increased in 2015 to 89.19% from 69.23% in 2014, then decreased to 57.14% again in 2016. A decreasing trend of resistance to ciprofloxacin, norfloxacin, and tetracyclines was found from 2014 to 2016. Among the macrolides, an increasing trend of resistance was found for azithromycin from 2014 (38.46%) to 2015 (72.97%), while it decreased in 2016 (42.86%). While about half of the ETEC strains were resistant to erythromycin during 2014–2015, all the strains were resistant in 2016. Trimethoprim/sulfamethoxazole resistance had a decreasing trend in 2014 (53.85%), followed by 2016 (42.86%) and 2015 (13.51%). An increase in resistance was observed for ceftriaxone and streptomycin from 2014 to 2016. Resistance to ampicillin remained the same over the years (Figure 5a).

In the environmental samples, among the macrolides, azithromycin showed increasing resistance from 2015 (15.38%) to 2016 (66.67%), and erythromycin showed a decreasing trend. A higher proportion of ETEC strains resistant to nalidixic acid and trimethoprim/sulfamethoxazole was observed over time from 2015 (15.38%) to 2016 (33.33%). Resistance was decreased for tetracycline, ceftriaxone, and ampicillin from 2015 to 2016. An increase in ETEC strains with intermediate susceptibility to streptomycin was found from 2015 (15.38%) to 2016 (33.33%) (Figure 5b).

### 3.8. AMR Pattern in Non-ETEC Strains

**Figure 6:**
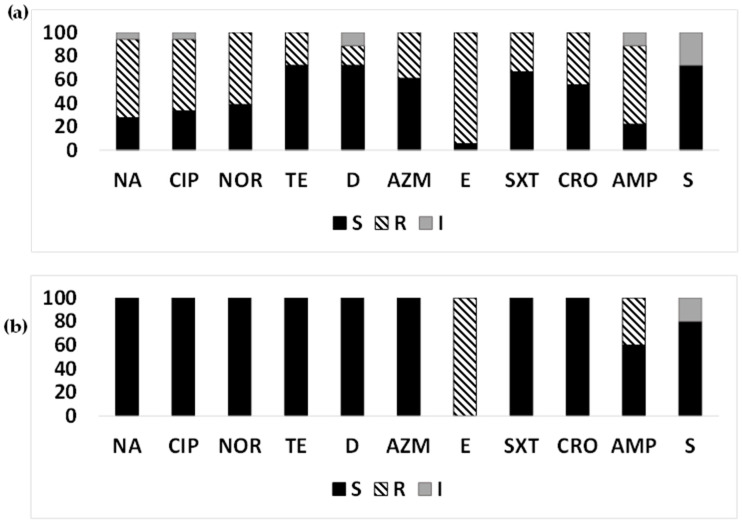
Antimicrobial susceptibility pattern of non-ETEC strains: The proportion of sensitive or resistant (**a**) non-ETEC strains isolated from diarrheal patients in Mathbaria and Chhatak; (**b**) non-ETEC strains isolated from surface water in Mathbaria and Chhatak. Black-filled bar: sensitive; Pattern-filled bar: Resistant; and Grey-filled bar: intermediate susceptibility. The x axis represents the antibiotic, and the y axis represents the percentage of sensitive and resistant strains.

Resistance to quinolone, tetracyclines, trimethoprim/sulfamethoxazole, ceftriaxone, and ampicillin among non-ETEC *E. coli* isolates from diarrheal patients was 61.1–66.7%, 16.7–27.8%, 33.3%, 44.4%, and 66.7%, respectively. About 38.9% of non-ETEC strains from diarrheal patients was resistant to azithromycin, and 94.4% strains showed resistance to erythromycin. All the environmental non-ETEC isolates were sensitive to azithromycin; however, 100% of the strains showed resistance to erythromycin. Resistance to ampicillin among non-ETEC *E. coli* isolates from environmental samples was 40%. Streptomycin showed intermediate susceptibility in both cases, which was 27.78% for diarrheal patients and 20% for environmental samples (Figure 6a,b).

Among the ETEC isolates from the PSF drinking water in Mathbaria, the highest proportion of ETEC was resistant to ampicillin (100%) followed by macrolides (87.5%), ceftriaxone (87.5%), tetracyclines (25%), and quinolones (12.5%).

## 4. Discussion

This is a unique study and, to our knowledge, the first study where the antibiotic sensitivity pattern of ETEC strains isolated from diarrheal patients and environmental sources were analyzed simultaneously over the years from two geographically distinct areas in Bangladesh. This study found a high prevalence of MDR ETEC strains in the diarrhea patients and environment from both Coastal Mathbaria and Inland Chhatak.

An interesting finding from this study was that, while a significantly high proportion of the ETEC strains was resistant to macrolides, a majority were susceptible to ciprofloxacin and norfloxacin, both from the patients and the environment in the two sites. Ciprofloxacin and norfloxacin were the drugs of choice in the 1990s for treating diarrheal pathogens in Bangladesh. Fluroquinolone resistance increased over the years [22], and azithromycin replaced quinolones in treating diarrheal patients. These changes in drug regimens over the years were reflected in the patterns of ETEC AMR found in this study. The overuse of azithromycin for the treatment of diarrheal and respiratory diseases in recent years may have contributed to the increase in resistance to azithromycin among ETEC isolates [23].

Notably, in the diarrheal patients, about half of the ETEC isolates in Mathbaria and about 90% in Chhatak were resistant to both azithromycin and erythromycin. However, in the aquatic environment, ETEC isolates were ~100% resistant to erythromycin while only 20 to 30% resistant to azithromycin. Azithromycin is used for many indications, including diarrheal, respiratory, and genitourinary diseases. Erythromycin was used for treating cholera, and its susceptibility decreased over the years from 98.5% in 2000 to 2005 to 0.96% in 2016–2021 [24]. The overuse of a drug favors the emergence of bacteria resistant to that drug, and these resistant bacteria can then be transferred to the environment through fecal contaminations. Limiting the use of the overused drug allows susceptible bacteria to recover. This reversion back to susceptibility was also noted for tetracycline and doxycycline, which were the drugs of choice for the treatment of cholera and were later, due to high resistance [25], replaced by azithromycin. In this study, ETEC strains from both patients and the environment were highly susceptible to tetracyclines.

Comparing the ETEC AMR profiles from the patients found in this study in 2014–2016 with the published data from Dhaka, the capital city in Central Bangladesh, from 2005 to 2009 [26], resistance to azithromycin increased by 23% in 2005–2009 to 60–100% in this study, while resistance to erythromycin decreased from 96% in 2005–2009 to 60% in Mathbaria and 90% in Chhatak. Resistance to ciprofloxacin and norfloxacin were lower in our study, while resistance to nalidixic acid (83%) in Dhaka was similar to Chhatak. Low-level resistance to the quinolone group of antibiotics was also reported among ETEC strains from Japan (1996), Egypt (1995–1998), and Peru (2006–2011) [27,28,29]. Resistance to tetracyclines was reduced from ~40% in 2005–2009 to <20% in our study. The reduced rate of resistance to tetracycline was reported in *V. cholerae* from India (2004–2013) [30].

Although our study sites, Chhatak and Mathbaria, are geographically different and far apart from each other, the AMR profiles observed for ETEC strains were overall similar with resistance to macrolides, ampicillin, and trimethoprim/sulfamethoxazole, which suggests similar types and frequencies of antibiotic use in these two places. A higher proportion of MDR-ETEC strains (72%), while less diverse (four resistant profiles), was found in diarrheal patients in Chhatak compared to 60% MDR ETEC in Mathbaria, with nine resistant profiles. By contrast, the highest percentage of MDR-ETEC (69%) with five resistance profiles was observed in Mathbaria surface water compared to Chhatak (30%), with two resistance profiles. This could be expected, as Mathbaria is in the coastal area, and coastal flooding is often noted, which increases the transmission of AMR between humans and surface water [31]. Another major difference noted between the sites was that, while 81% of the ETEC strains from the patients were resistant to nalidixic acid in Mathbaria, less than 20% strains were resistant in Chhatak, although 40% showed intermediate resistance, which could be changed to either resistant or susceptible in the future. In addition, higher proportions of isolates were resistant to macrolides in Chhatak than Mathbaria. More ETEC isolates from water were resistant to ceftriaxone in Mathbaria than in Chhatak, which may suggest less use of the drug here.

Although the proportion of the resistant ETEC strains was lower in the surface water than that of the diarrhea patients in each site, the overall AMR pattern was, however, similar, which indicates high fecal contamination and the transmission of diarrheal pathogens through the surface water. The major difference of ETEC AMR between patients and surface water in Mathbaria was a higher proportion of ETEC strains resistant to erythromycin, ceftriaxone, and ampicillin and fewer resistant to nalidixic acid, azithromycin, trimethoprim/sulfamethoxazole, and streptomycin in the surface water than in the patients. In Chhatak, a majority (~80%) of the ETEC strains from surface water were susceptible to streptomycin; ~80% of the strains from patients were intermediately resistant, which may change to fully resistant in the future. Trimethoprim/sulfamethoxazole, azithromycin, and ampicillin had higher—and nalidixic acid had lower—resistance in patients than in the surface water.

An interesting observation was that the patterns of AMR in ETEC varied by the toxin types depending on the type of antibiotic. Overall, LT-ETEC and LT+ST-ETEC were more resistant to antibiotics than ST-ETEC. Among patient isolates, a higher proportion of LT-ETEC and LT+ST-ETEC strains were resistant to nalidixic acid, trimethoprim/sulfamethoxazole, and ampicillin than ST-ETEC strains. While ST-ETEC and LT+ST-ETEC are more associated with moderate to severe diarrhea, LT-ETEC was found in similar proportion both among the asymptomatic and diarrhea cases [3]. Due to frequent and long colonization in the intestine with other bacteria among the asymptomatic cases, a possible cause is that LT-ETEC may acquire resistance. As in the patients, in the surface water, the resistance to nalidixic acid was higher in LT-ETEC and LT+ST-ETEC than ST-ETEC; however, LT+ST-ETEC had a higher resistance to ciprofloxacin and norfloxacin, while having a lower resistance to macrolides.

In our study, ETEC MDR strains were highly prevalent in drinking water, and the majority of the ETEC strains were resistant to ampicillin, macrolides, and ceftriaxone. Of note, the drinking water samples that were tested from Mathbaria were from sand filters, which are intended to be safe. Tap water contaminated with ETEC was reported before from the Dhaka city in Bangladesh [32]. There appeared to be a lower proportion of resistance in the non-ETEC *E. coli* compared to ETEC, which may suggest that pathogenic ETEC strains are capable of acquiring resistance faster.

Non-ETEC strains isolated from diarrheal patients showed a reduced level of resistance to quinolone, tetracycline, and trimethoprim/sulfamethoxazole compared to ETEC strains. Both non-ETEC and ETEC strains isolated from diarrheal patients had similar levels of resistance to the antibiotic’s macrolides, ceftriaxone, and ampicillin. Compared to ETEC strains, most of the non-ETEC strains isolated from surface water were sensitive to the majority of the antibiotics tested. Non-ETEC and ETEC from surface water showed similar resistance patterns to erythromycin.

Over the years, from 2014 to mid-2016, the major trend in changes in AMR showed an increase in ETEC strains that are sensitive to ciprofloxacin, norfloxacin, tetracycline, and doxycycline. Resistance to azithromycin and ceftriaxone and streptomycin increased in the patients. In the surface water, resistance to nalidixic acid, azithromycin, and trimethoprim/sulfamethoxazole increased, while erythromycin and ceftriaxone decreased. These patterns in AMR may suggest that the ETEC strains in the patients acquire resistance first and are then transmitted to the environment.

This study has many strengths, which includes monitoring the AMR of ETEC simultaneously in patients and aquatic water bodies throughout the year. We compared the AMR of ETEC in two geographically distinct sites. We used sample processing and microbiological methods, which maximize the isolation of ETEC strains from the aquatic environment. The study also has limitations. We conducted the surveillance for only one year in Chhatak and were therefore not able to compare the AMR trends between years. Due to the small sample size for each sample type, not enough statistical power was achieved to analyze for significance.

## 5. Conclusions

This study found an alarmingly high prevalence of MDR ETEC strains in patients and the aquatic environment in both the study sites in Bangladesh. The overall similar AMR pattern between patients and surface water suggests high risks for the transmission of AMR strains between humans and the environment. The ETEC strains were resistant to the most common antibiotics currently used to treat patients with diarrhea, which is alarming and needs more attention. The relation between the changes in the AMR patterns (resistant or susceptible) with the overuse or limited use of antibiotics suggests that a close monitoring of antibiotic resistance in humans and the environment is crucial, and guidelines are needed to restrict the use of resistant antibiotics for a period, which may reverse the antibiotic back to sensitive again, allowing them to be used effectively. We are currently analyzing antibiotic-resistant genes among the clinical and environmental ETEC isolates via whole genome sequencing to understand this transmission.

## Figures and Tables

**Table 1:** Number of ETEC strains tested for AMR from Mathbaria and Chhatak.

	Mathbaria	Chhatak
Patients	57	29
Surface water	15	10
Pond sand filter (drinking water)	8	
Sediment	3	
Plankton	0	2
Totals	83	41

**Table 2:** Resistance profiles of MDR-ETEC strains isolated from Mathbaria and Chhatak diarrheal patients.

Location	Type	Resistance Profile	% Of Strains
Mathbaria	I	T, M, SXT, CRO, AMP, S	14.7
	II	Q, T, SXT, CRO, AMP, S	2.9
	III	Q, T, SXT, CRO, AMP	5.88
	IV	Q, T, M, SXT, AMP	2.9
	V	Q, M, CRO, AMP	23.53
	VI	M, SXT, CRO, AMP	5.88
	VII	Q, M, AMP	29.41
	VIII	M, SXT, AMP	11.76
	IX	Q, M, S	2.9
Chhatak	I	Q, T, M	4.76
	II	M, CRO, AMP	9.5
	III	M, SXT, AMP	71.43
	IV	Q, M, AMP	14.3

Q, quinolone; T, tetracyclines; M, macrolide; SXT, trimethoprim/sulfamethoxazole; CRO, ceftriaxone; AMP, ampicillin; S, streptomycin.

**Table 3:** Resistance profiles of MDR-ETEC strains isolated from Mathbaria and Chhatak surface water.

Location	Type	Resistance Profile	% of Strains
Mathbaria	I	T, M, AMP	9.1
	II	M, CRO, AMP	63.6
	III	Q, M, CRO, AMP	9.1
	IV	T, M, CRO, AMP	9.1
	V	Q, M, SXT, AMP	9.1
Chhatak	I	M, CRO, AMP	33.33
	II	Q, M, AMP	66.67

Q, quinolone; T, tetracyclines; M, macrolide; SXT, trimethoprim/sulfamethoxazole; CRO, ceftriaxone; AMP, ampicillin.

## Data Availability

Data are contained within the article.
